# Supplementary material for: Influenza A virus reassortment in mammals gives rise to genetically distinct within-host subpopulations
Source: Nat Commun. 2022 Nov 11;13:6846. doi: 10.1038/s41467-022-34611-z (PMC9652339; doi:10.1038/s41467-022-34611-z)
Supplement: Supplementary file 1 — Supplementary Information [file 41467_2022_34611_MOESM1_ESM.pdf]

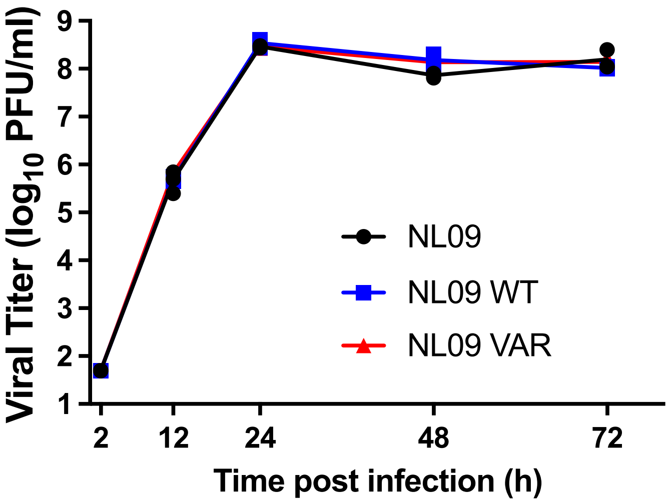

Supplementary Fig. 1. NL09 WT and NL09 VAR show comparable replication in MDCK cells. Viral replication from low MOI was monitored over time in three replicate culture dishes. Mean and standard deviation are plotted.

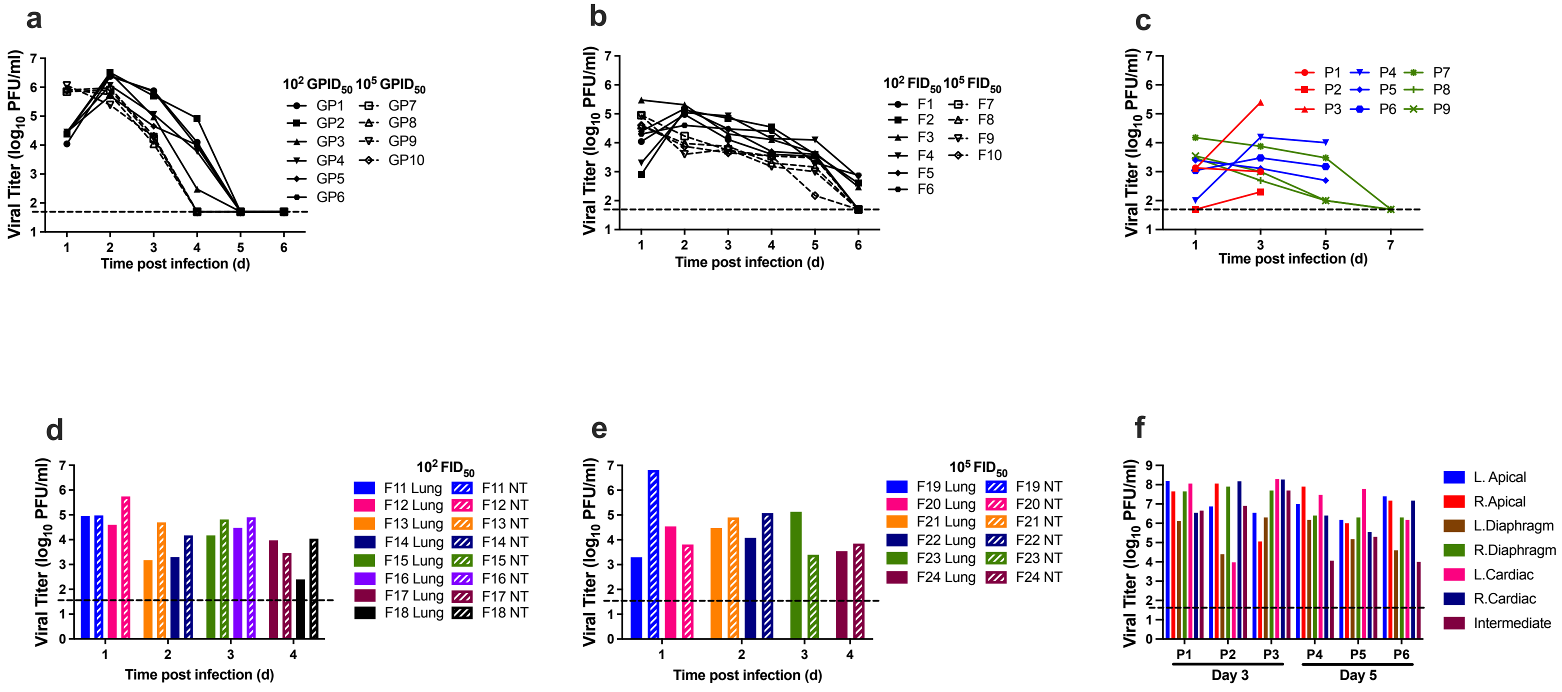

Supplementary Fig. 2. Viral loads in infected guinea pigs, ferrets and swine. Viral shedding determined by plaque assay from guinea pig nasal washes (A), ferret nasal washes (B), swine nasal swabs (C), ferret nasal turbinates (D), ferret lung homogenates (E), and swine lung homogenates (F) over time are shown. The horizontal dashed line represents the limit of detection of the plaque assay (50 PFU/mL).

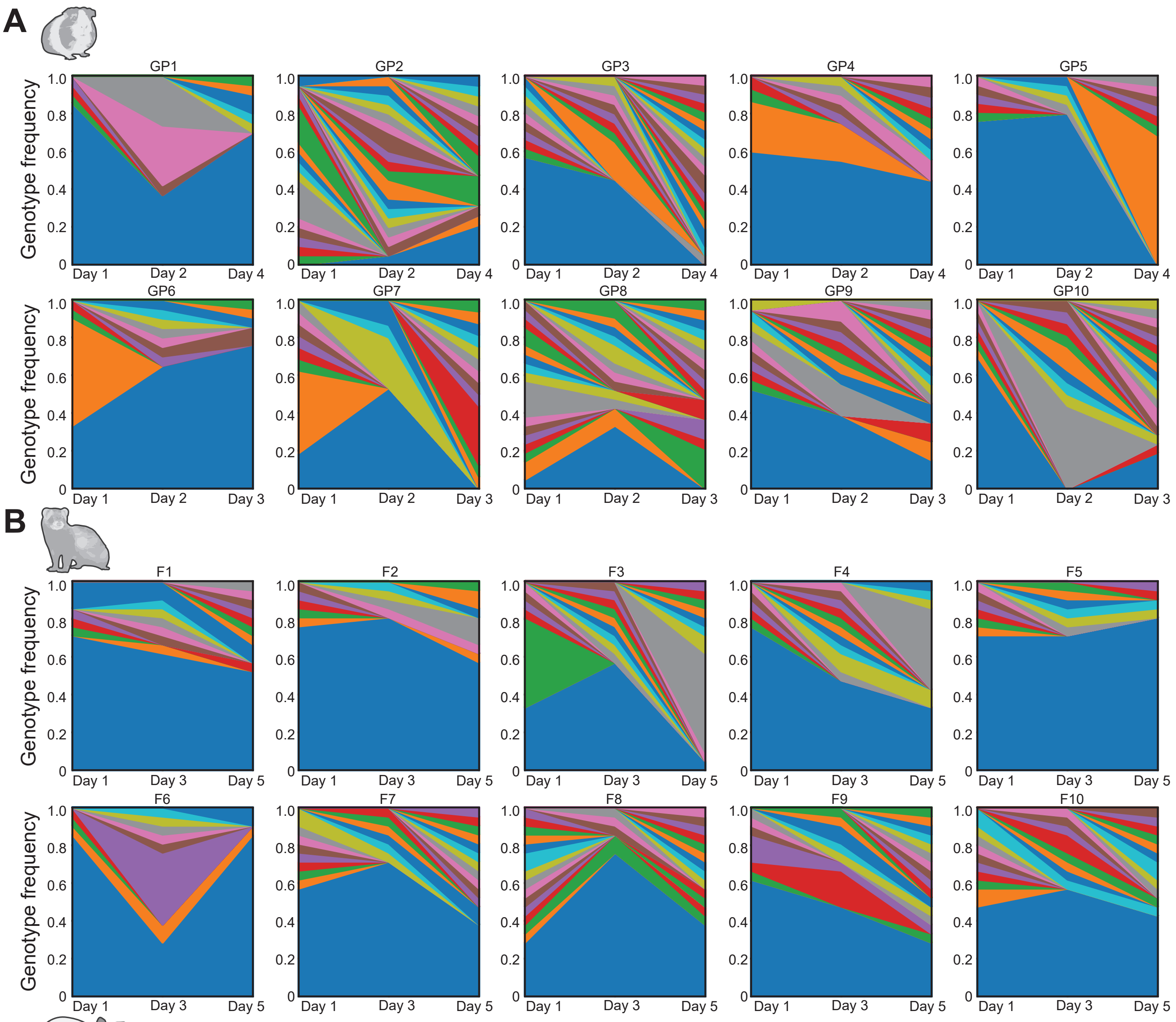

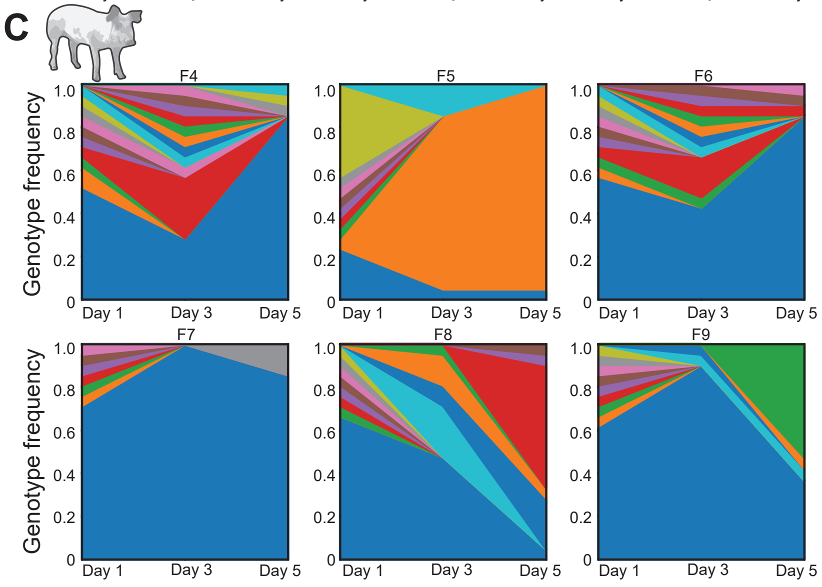

Supplementary Fig. 3. Viral genotypic diversity generated through reassortment in the mammalian nasal tract.(Corresponds to Figure 1) Stacked plots showing frequencies of unique genotypes detected in guinea pigs (A), ferrets (B) or swine (C) over time. The sections in blue and orange represent the WT and VAR parental genotypes, respectively. Animal silhouettes were generated using Biorender.com

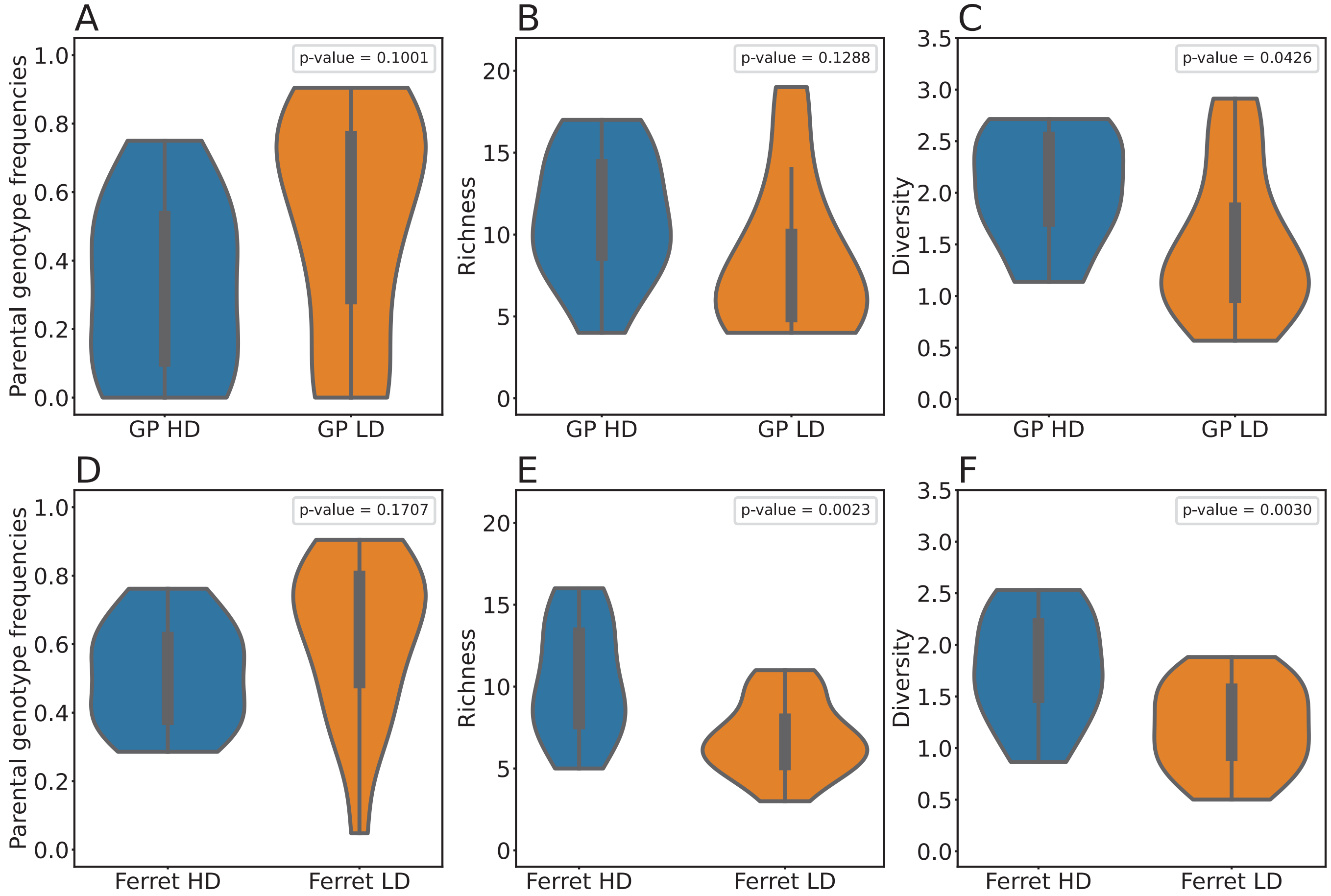

Supplementary Fig. 4. Effects of inoculation dose on viral diversity generated through reassortment in the nasal tract. (Corresponds to Figure 1) Results from guinea pigs are shown in panels A–C, ferrets in panels D–F. HD indicates high dose ($1x10^5$ ID50) and LD indicates low dose ($1x10^2$ ID50) groups. The distribution of parental genotype frequencies (A, D), richness (B, E) and diversity (C, F) across all time points in each species is shown with violin plots. P values were determined by ANOVA.

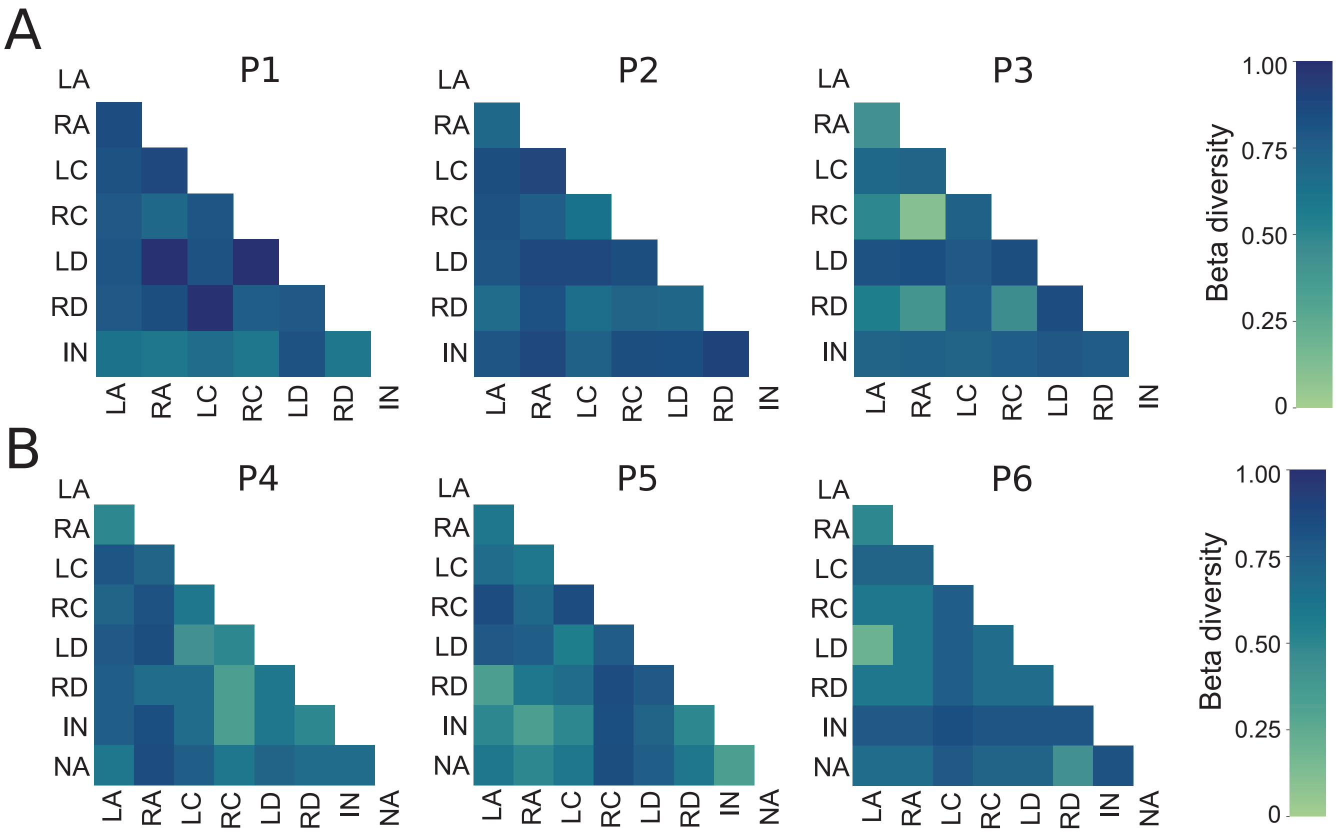

Supplementary Fig. 5. Reassortant viral populations show extensive compartmentalization within the swine respiratory tract. (Corresponds to Figure 5) Heat maps showing beta diversity of viral populations in the lung lobes and nasal tract of individual pigs. Pig ID number is indicated above each matrix. (A) Tissues were extracted on day 3 post-inoculation. (B) Tissues were extracted on day 5 post-inoculation. The tissue sites are abbreviated as NA-Nasal; LA- Left Apical; RA- Right Apical; LC-Left Cardiac; RC-Right Cardiac; LD-Left Diaphragmatic; RD-Right Diaphragmatic; IN-Intermediate.

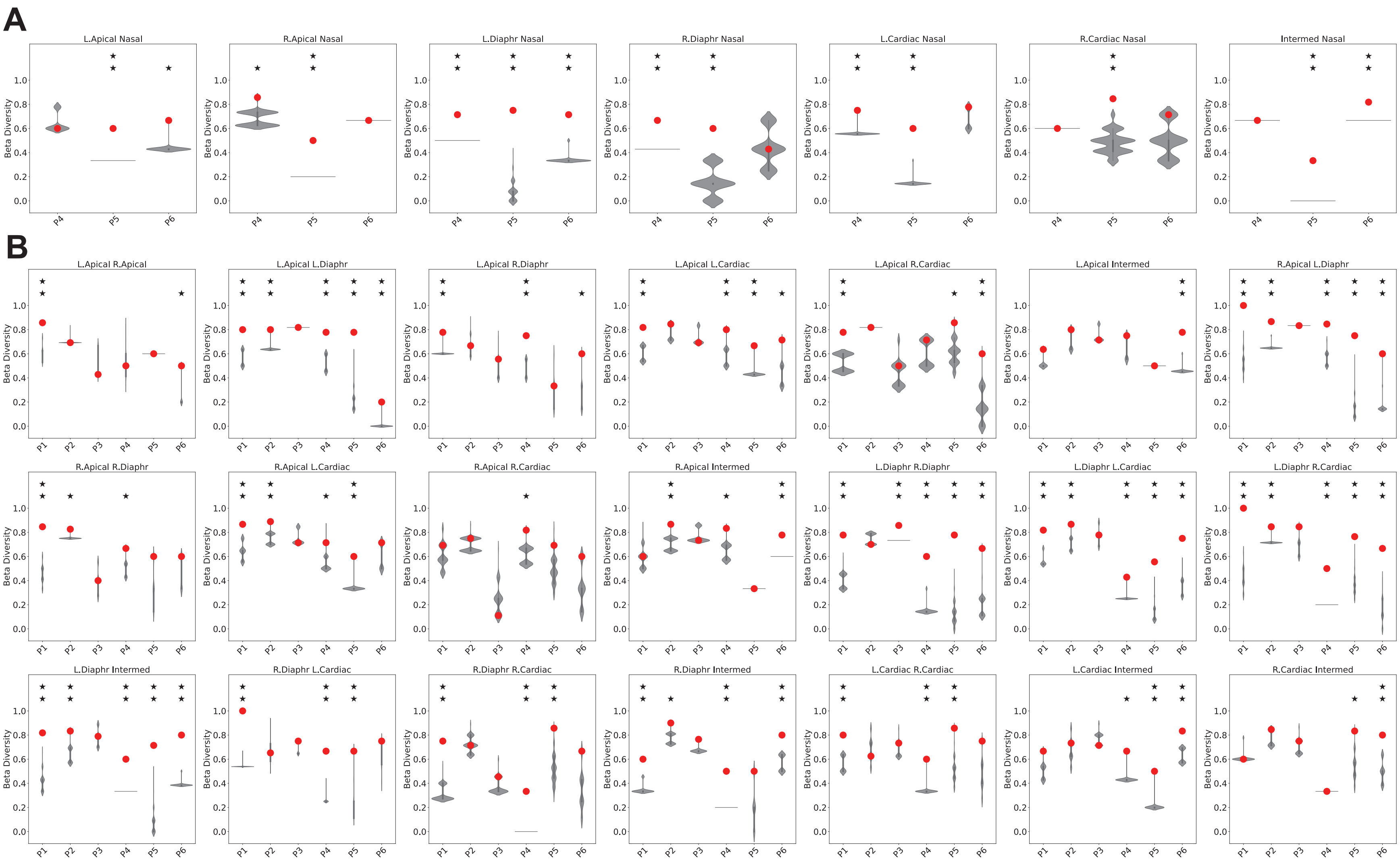

Supplementary Fig. 6. Reassortant viral populations show extensive compartmentalization within the swine respiratory tract. (Corresponds to Figure 5) Normalized beta diversity between each lung lobe and nasal tract (A) and between lung lobes (B) are plotted with observed results (colored points) overlaid on the distribution of simulated data (gray violins). One star indicates that observed data is above the 95th percentile of the distribution; two stars indicates that observed data is above the 99th percentile

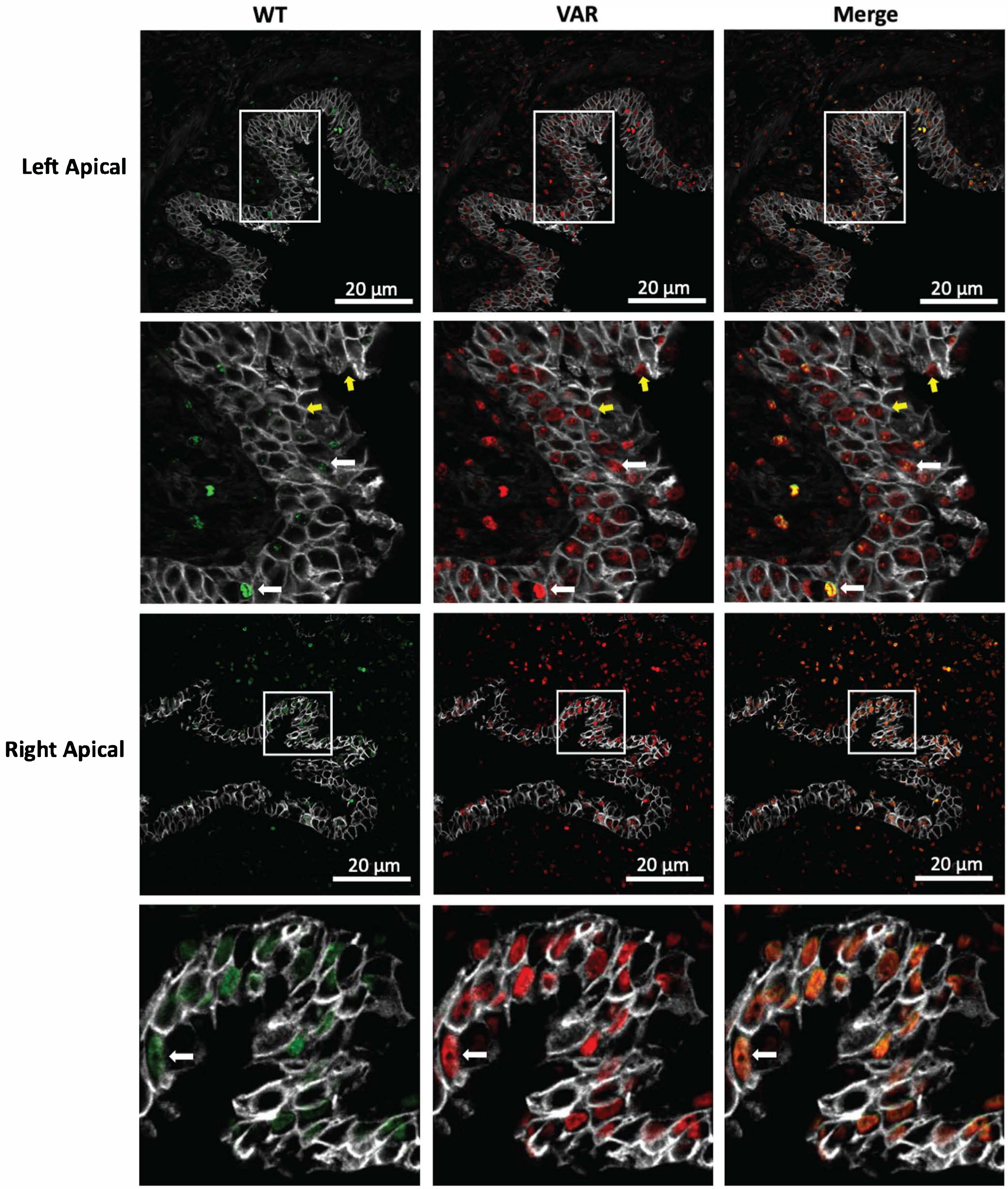

Supplementary Fig. 7. Coinfection is common in the swine respiratory tract. (Corresponds to Figure 5) Immunohistochemistry images of lung tissue sections stained for WT (green) and VAR (red) viruses at day 3 post-inoculation. The lung lobe sampled is indicated at the left. Gray staining marks epithelial cell borders. Yellow coloring in merged images indicates the presence of both WT and VAR HA antigens in the same cell. Zoomed insets are shown with white arrows indicating co-infected cells and yellow arrows indicating singly infected cells.

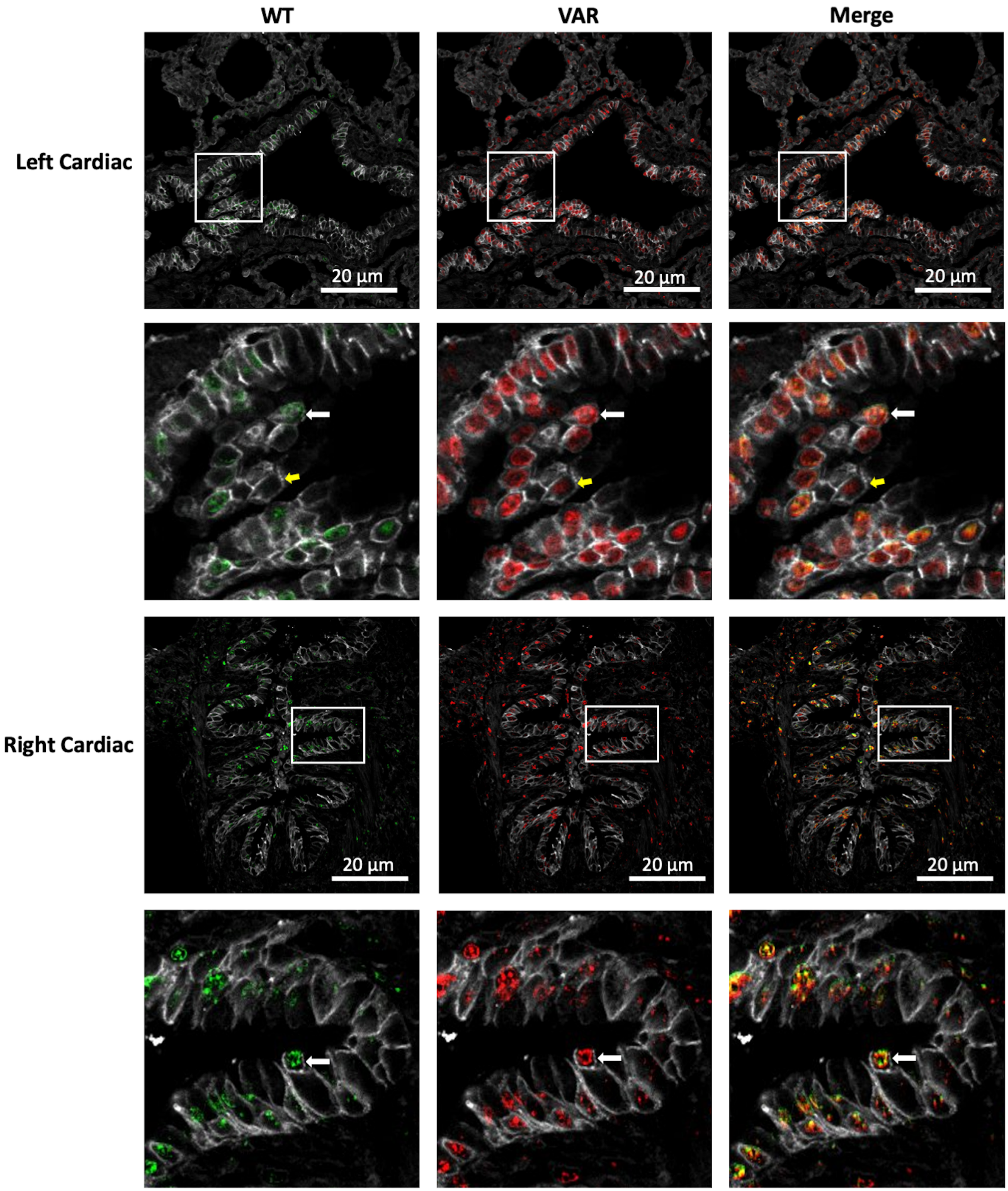

WT
VAR
Merge
Left Cardiac
20 µm
20 µm
20 µm
Right Cardiac
20 µm
20 µm
20 µm

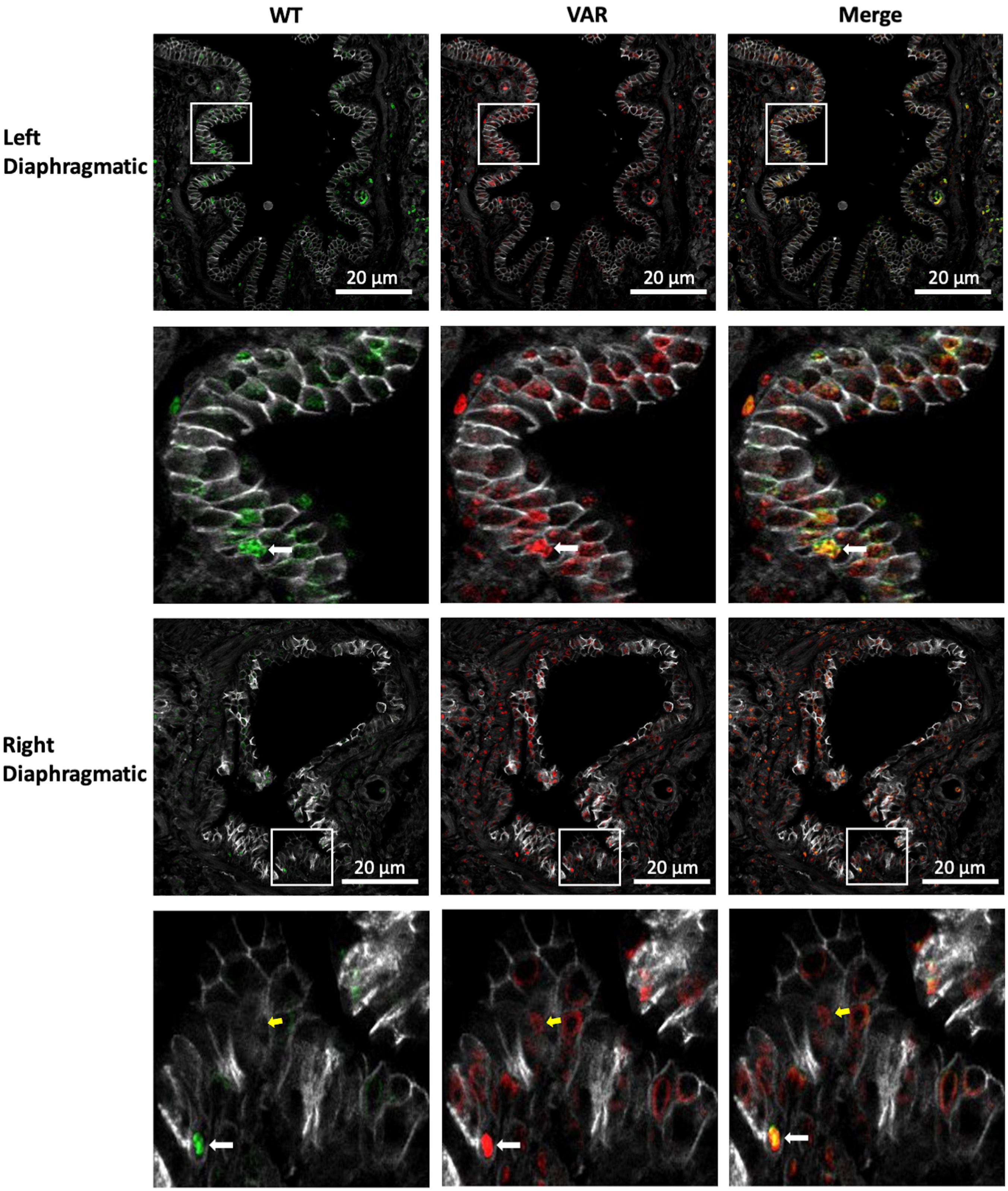

WT
VAR
Merge
Left Diaphragmatic
Right Diaphragmatic
20 µm
20 µm
20 µm
20 µm
20 µm
20 µm

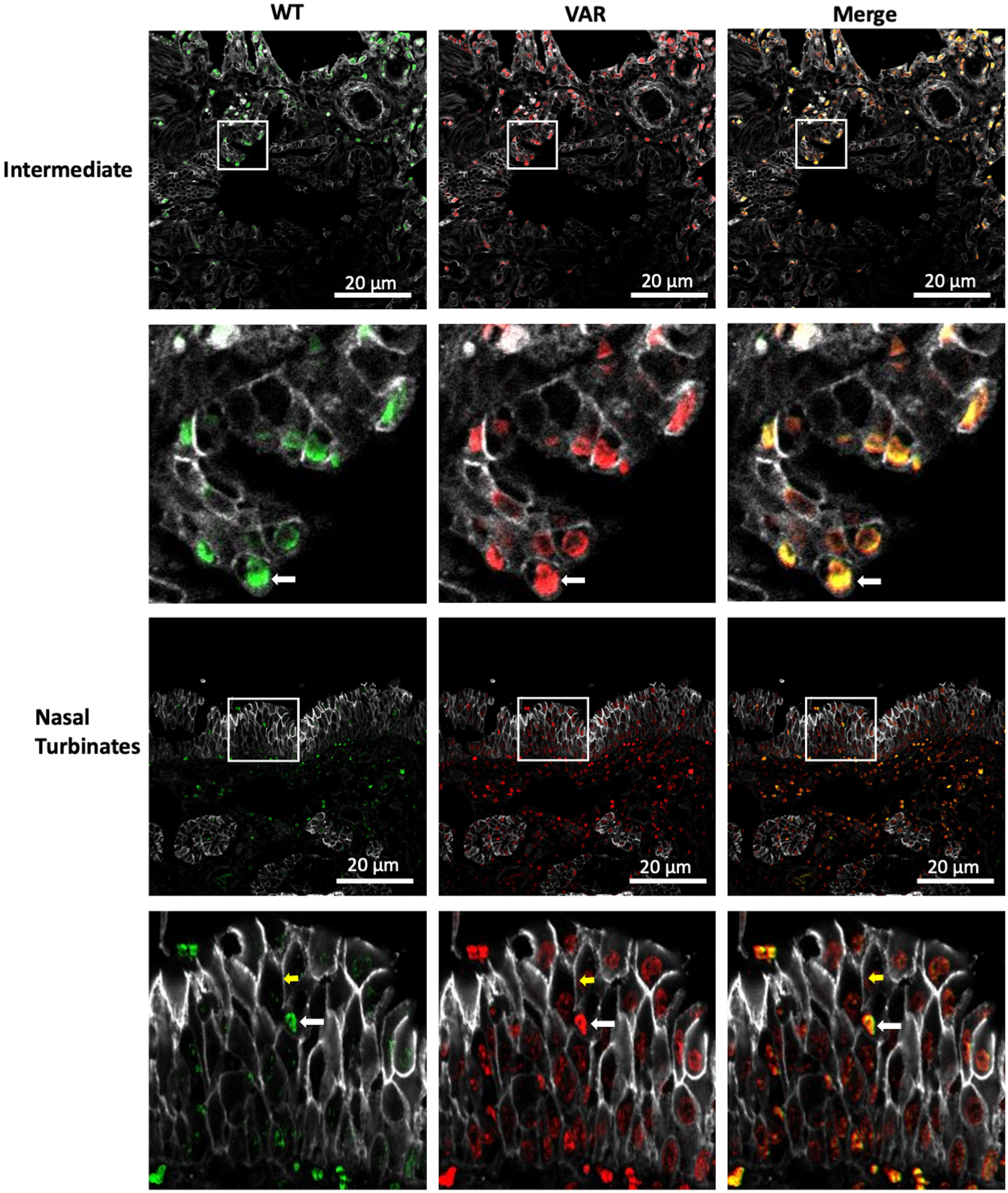

WT
VAR
Merge
Intermediate
Nasal
Turbinates
20 µm
20 µm
20 µm
20 µm
20 µm
20 µm

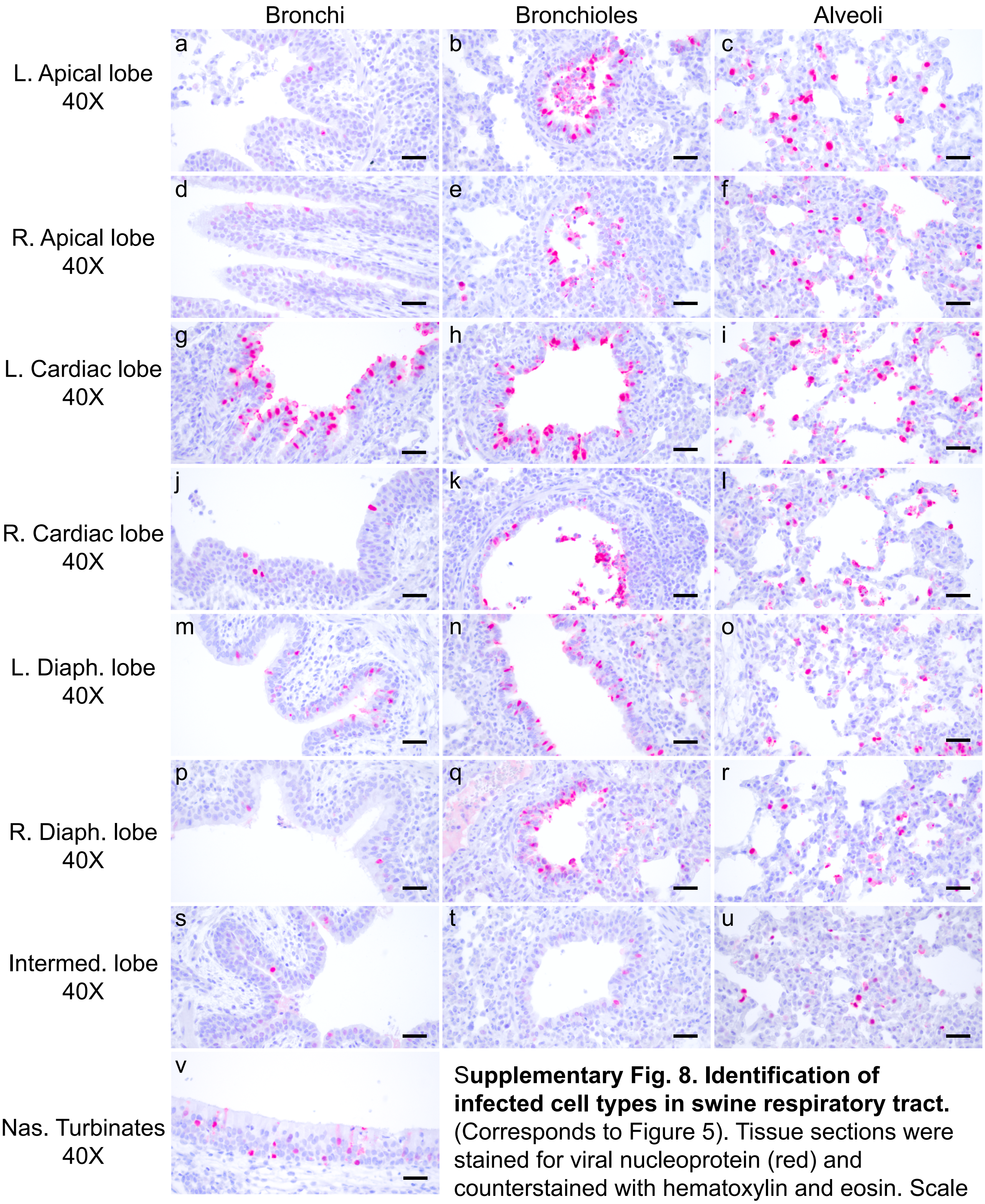

Supplementary Fig. 8. Identification of infected cell types in swine respiratory tract. (Corresponds to Figure 5). Tissue sections were stained for viral nucleoprotein (red) and counterstained with hematoxylin and eosin. Scale bars are 20 µm.

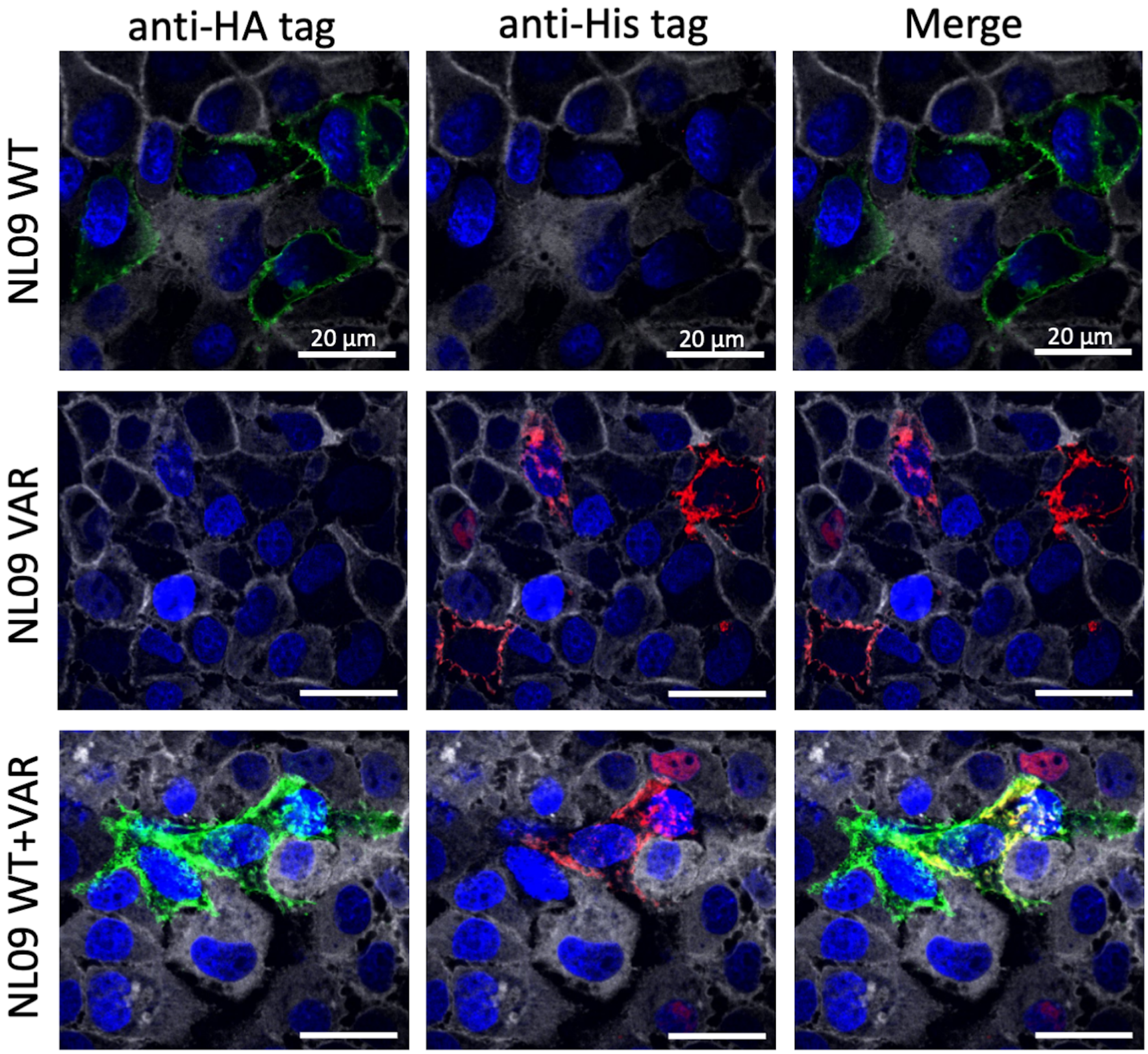

Supplementary Fig. 9. Validation of the antibodies used for immunohistochemistry. Immunofluorescence images of MDCK cells stained for WT (green) and VAR (red) viruses at 24 h post infection. Gray staining marks epithelial cell borders. Yellow coloring in merged images indicates the presence of both WT and VAR HA antigens in the same cell.

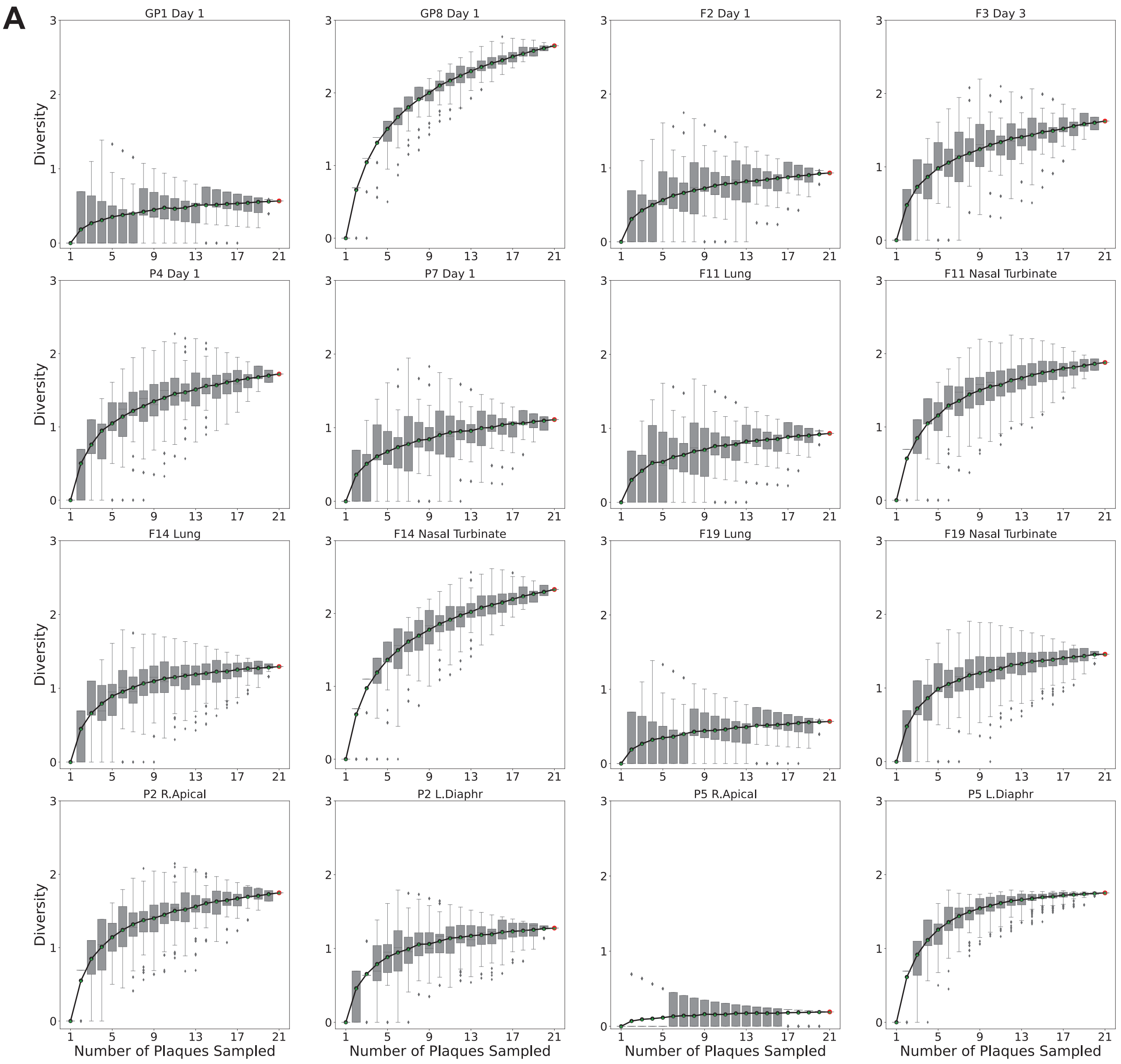

Supplementary Fig. 10. Evaluation of the sensitivities of diversity and beta diversity to the number of viral plaques analyzed. The impact of the number of plaques analyzed on the detection of diversity in guinea pigs, pigs and ferrets (A), beta diversity in ferrets (B) and beta diversity in pigs (C) was assessed by sub-sampling from our experimental dataset with 1000 replicate simulations performed at each number of plaques. Representative samples were analyzed and the sample is indicated above each facet. Points with the green triangles inside indicate the mean. Boxes represent the first and third quartiles, with the middle line representing the median. Whiskers show the minimum and maximum of the data with the outliers omitted outside of two standard deviations (plotted by the diamonds). The red data point shows the observed result reported in the main figures

B

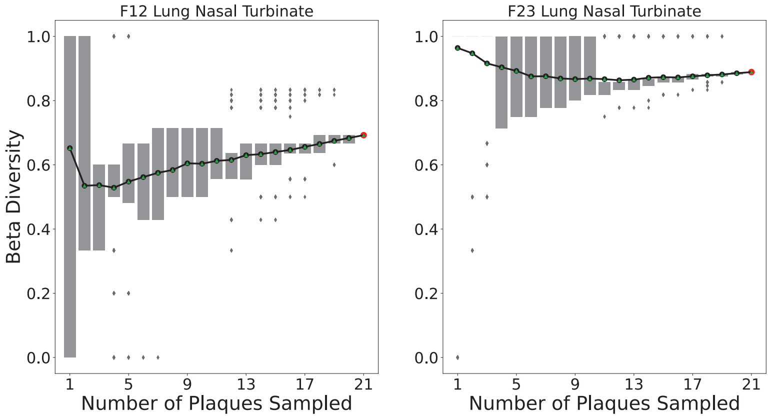

Supplementary Fig. 10. Evaluation of the sensitivities of diversity and beta diversity to the number of viral plaques analyzed. The impact of the number of plaques analyzed on the detection of diversity in guinea pigs, pigs and ferrets (A), beta diversity in ferrets (B) and beta diversity in pigs (C) was assessed by sub-sampling from our experimental dataset with 1000 replicate simulations performed at each number of plaques. Representative samples were analyzed and the sample is indicated above each facet. Points with the green triangles inside indicate the mean. Boxes represent the first and third quartiles, with the middle line representing the median. Whiskers show the minimum and maximum of the data with the outliers omitted outside of two standard deviations (plotted by the diamonds). The red data point shows the observed result reported in the main figures

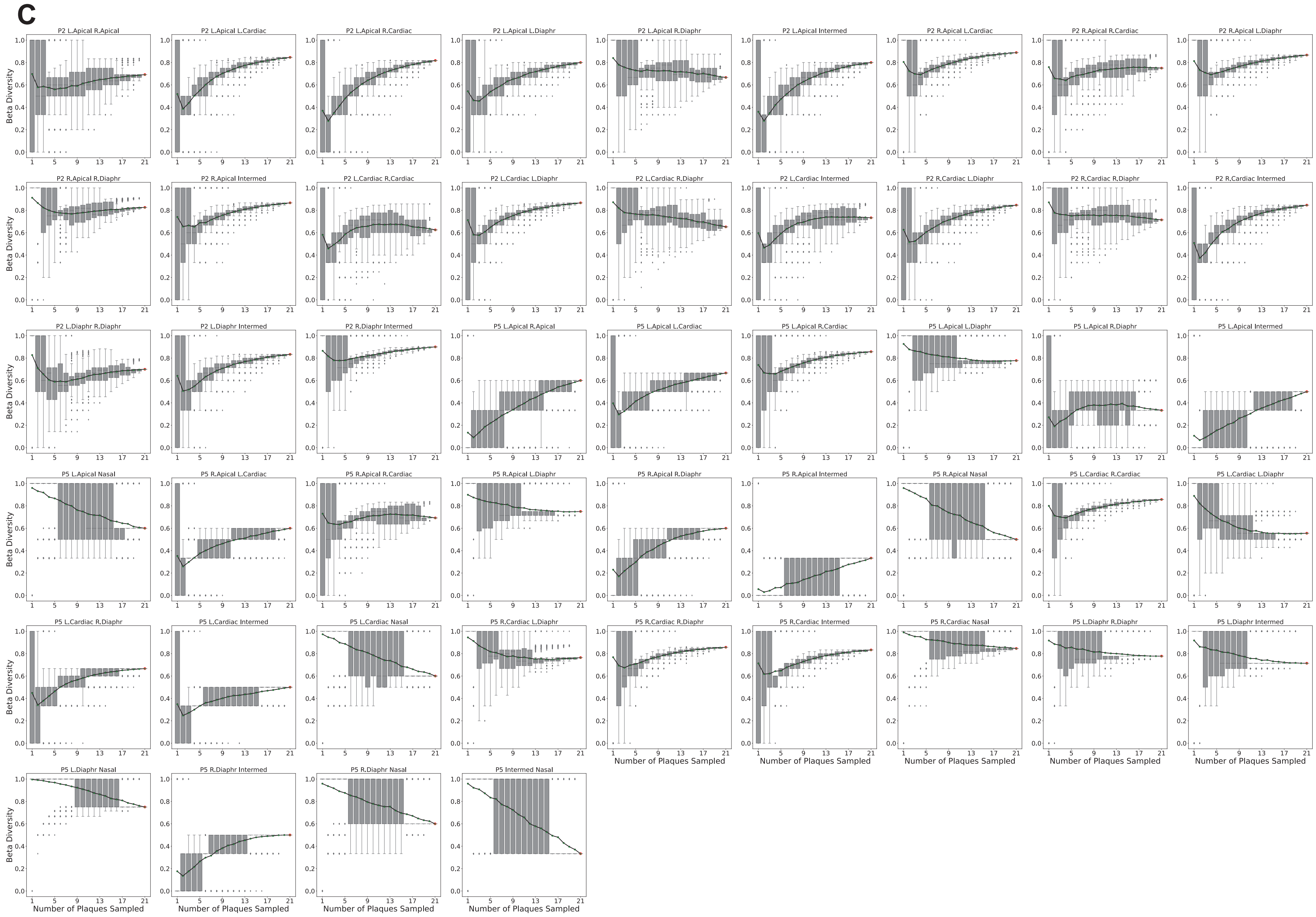

Supplementary Fig. 10. Evaluation of the sensitivities of diversity and beta diversity to the number of viral plaques analyzed. The impact of the number of plaques analyzed on the detection of diversity in guinea pigs, pigs and ferrets (A), beta diversity in ferrets (B) and beta diversity in pigs (C) was assessed by sub-sampling from our experimental dataset with 1000 replicate simulations performed at each number of plaques. Representative samples were analyzed and the sample is indicated above each facet. Points with the green triangles inside indicate the mean. Boxes represent the first and third quartiles, with the middle line representing the median. Whiskers show the minimum and maximum of the data with the outliers omitted outside of two standard deviations (plotted by the diamonds). The red data point shows the observed result reported in the main figures
