# Supplementary material for: Influenza A virus reassortment in mammals gives rise to genetically distinct within-host subpopulations
Source: Nat Commun. 2022 Nov 11;13:6846. doi: 10.1038/s41467-022-34611-z (PMC9652339; doi:10.1038/s41467-022-34611-z)
Supplement: Supplementary file 3 — Description of Additional Supplementary Files [file 41467_2022_34611_MOESM3_ESM.pdf]

Description of Additional Supplementary Files

File Name: Supplementary Data 1
Description: . **Genotype tables.** Viral genotypes detected in guinea pigs, ferrets and swine are included. Each table corresponds to a biological sample and each row in each table corresponds to a single viral isolate. The columns show the eight viral gene segments. Blue color blocks indicate WT genes while red color blocks indicate VAR genes.

File Name: Supplementary Data 2
Description: **Identification of viral nucleoprotein positive cells present in ferret lung, pig lung and pig nasal turbinate.**

File Name: Supplementary Data 3
Description: **Primer sequences for NL09 WT/VAR HRM analysis**
