# Supplementary material for: Influenza A virus reassortment in mammals gives rise to genetically distinct within-host subpopulations
Source: Nat Commun. 2022 Nov 11;13:6846. doi: 10.1038/s41467-022-34611-z (PMC9652339; doi:10.1038/s41467-022-34611-z)
Supplement: Supplementary file 7 — Reporting Summary [file 41467_2022_34611_MOESM7_ESM.pdf]

nature portfolio

Corresponding author(s): Lowen

Last updated by author(s): Oct 16, 2022

Reporting Summary

Nature Portfolio wishes to improve the reproducibility of the work that we publish. This form provides structure for consistency and transparency in reporting. For further information on Nature Portfolio policies, see our Editorial Policies and the Editorial Policy Checklist.

Statistics

For all statistical analyses, confirm that the following items are present in the figure legend, table legend, main text, or Methods section.

n/a	Confirmed	
☐	☒	The exact sample size (*n*) for each experimental group/condition, given as a discrete number and unit of measurement
☐	☒	A statement on whether measurements were taken from distinct samples or whether the same sample was measured repeatedly
☐	☒	The statistical test(s) used AND whether they are one- or two-sided<br>*Only common tests should be described solely by name; describe more complex techniques in the Methods section.*
☒	☐	A description of all covariates tested
☐	☒	A description of any assumptions or corrections, such as tests of normality and adjustment for multiple comparisons
☐	☒	A full description of the statistical parameters including central tendency (e.g. means) or other basic estimates (e.g. regression coefficient) AND variation (e.g. standard deviation) or associated estimates of uncertainty (e.g. confidence intervals)
☐	☒	For null hypothesis testing, the test statistic (e.g. *F*, *t*, *r*) with confidence intervals, effect sizes, degrees of freedom and *P* value noted<br>*Give P values as exact values whenever suitable.*
☒	☐	For Bayesian analysis, information on the choice of priors and Markov chain Monte Carlo settings
☒	☐	For hierarchical and complex designs, identification of the appropriate level for tests and full reporting of outcomes
☒	☐	Estimates of effect sizes (e.g. Cohen's *d*, Pearson's *r*), indicating how they were calculated

Our web collection on statistics for biologists contains articles on many of the points above.

Software and code

Policy information about availability of computer code

Data collection	Quantitative PCR data were collected using CFX Manager Software v2.1 (Bio-Rad) and Precision Melt Analysis Software v1.2 (Bio-Rad)
Data analysis	Figures were generated using Python 3 v3.10 and the packages matplotlib v3.6.0, NumPy v1.23.3, pandas v1.5.0 and seaborn v0.12.0. Simulations were conducted in Python 3 v3.10. Percentiles were calculated using the percentileofscore method from the SciPy package v1.9.1. Paired t-tests and ANOVA tests were performed using the ttest_rel method and the f_oneway method respectively from the SciPy Package v1.9.1. Code used for data analysis and simulations is available at: https://github.com/maxbagga/Influenza-A-virus-reassortment-in-mammals-gives-rise-to-spatially-distinct-sub-populations

For manuscripts utilizing custom algorithms or software that are central to the research but not yet described in published literature, software must be made available to editors and reviewers. We strongly encourage code deposition in a community repository (e.g. GitHub). See the Nature Portfolio guidelines for submitting code & software for further information.

Data

Policy information about availability of data

All manuscripts must include a data availability statement. This statement should provide the following information, where applicable:

- Accession codes, unique identifiers, or web links for publicly available datasets
- A description of any restrictions on data availability
- For clinical datasets or third party data, please ensure that the statement adheres to our policy

Raw data used for the generation of Figures 1-5 and Supplementary Figures 1-10 are included as Source Data and in Supplementary Table 1-3.

Human research participants

Policy information about studies involving human research participants and Sex and Gender in Research.

Reporting on sex and gender	*Use the terms sex (biological attribute) and gender (shaped by social and cultural circumstances) carefully in order to avoid confusing both terms. Indicate if findings apply to only one sex or gender; describe whether sex and gender were considered in study design whether sex and/or gender was determined based on self-reporting or assigned and methods used. Provide in the source data disaggregated sex and gender data where this information has been collected, and consent has been obtained for sharing of individual-level data; provide overall numbers in this Reporting Summary. Please state if this information has not been collected. Report sex- and gender-based analyses where performed, justify reasons for lack of sex- and gender-based analysis.*
Population characteristics	*Describe the covariate-relevant population characteristics of the human research participants (e.g. age, genotypic information, past and current diagnosis and treatment categories). If you filled out the behavioural & social sciences study design questions and have nothing to add here, write "See above."*
Recruitment	*Describe how participants were recruited. Outline any potential self-selection bias or other biases that may be present and how these are likely to impact results.*
Ethics oversight	*Identify the organization(s) that approved the study protocol.*

Note that full information on the approval of the study protocol must also be provided in the manuscript.

Field-specific reporting

Please select the one below that is the best fit for your research. If you are not sure, read the appropriate sections before making your selection.

☒ Life sciences ☐ Behavioural & social sciences ☐ Ecological, evolutionary & environmental sciences

For a reference copy of the document with all sections, see nature.com/documents/nr-reporting-summary-flat.pdf

Life sciences study design

All studies must disclose on these points even when the disclosure is negative.

Sample size	Sample size for animal experiments (guinea pigs, n=10; ferrets, n=24; swine, n=9) was selected based on variance observed in prior experiments of a similar nature, as well as practical considerations of cost and space (Phipps et al. Nat Micro 2020; Ganti, Bagga et al. Viruses 2021). For determination of infectious dose, practical considerations for number of animals tested included cost and space constraints.
Data exclusions	In high resolution melt analysis to determine viral genotypes, if qPCR technical replicates yielded discordant results, the genotype for that gene was excluded. If one or more genes from a given isolate yielded ambiguous results, the full isolate was excluded from the dataset. These exclusion criteria were pre-established.
Replication	All animal experiments were performed once with multiple animals which act as biological replicates. For immnuohistochemistry imaging, images from four fields were taken for each sample from which representative images were chosen. Growth curves for NL09 WT and VAR viruses were repeated in three replicate wells.
Randomization	No formal randomization was used for animal studies because each experiment only contained one treatment group (e.g. NL09 infected guinea pigs). All animals were age and sex matched.
Blinding	Blinding was not performed because the assays used in our study require the knowledge of the virus strain and dose present in our sample.

Reporting for specific materials, systems and methods

We require information from authors about some types of materials, experimental systems and methods used in many studies. Here, indicate whether each material, system or method listed is relevant to your study. If you are not sure if a list item applies to your research, read the appropriate section before selecting a response.

Materials & experimental systems

n/a	Involved in the study
☐	☒ Antibodies
☐	☒ Eukaryotic cell lines
☒	☐ Palaeontology and archaeology
☐	☒ Animals and other organisms
☒	☐ Clinical data
☒	☐ Dual use research of concern

Methods

n/a	Involved in the study
☒	☐ ChIP-seq
☒	☐ Flow cytometry
☒	☐ MRI-based neuroimaging

Antibodies

Antibodies used	Mouse anti-HA Alexa Fluor 488 (Invitrogen catalogue number A-21287; clone 16B12); 1:50 dilution<br>Mouse ant-His Alexa Fluor 555 (Invitrogen catalogue number MA1-135-A555; clone 4E3D10H2/E3); 1:50 dilution<br>Rabbit anti-Na+K+ATPase Alexa Fluor 647 (Abcam catalogue number 198367; clone EP1845Y); 1:100 dilution<br>Goat anti-influenza NP polyclonal antibody (Abcam catalogue number 155877); 1:1000 dilution<br>Rabbit anti-goat biotinylated IgG (Vector laboratories catalogue number BA-5000); 1:5000 dilution
Validation	Specificity of HA Alexa Fluor 488 and His Alexa Fluor 555 antibodies for infected cells with relevant epitopes was verified by staining uninfected cells as a negative control. Cross reactivity between these antibodies for the alternate epitope was tested by reciprocal staining of cells infected with His-only or HA-only encoding viruses (Supp Fig 5). All antibodies were procured from commercial sources and have been independently validated by the manufacturers.

Eukaryotic cell lines

Policy information about cell lines and Sex and Gender in Research

Cell line source(s)	Madin-Darby canine kidney (MDCK) cells (ATCC), a gift from Dr. Robert Webster, St. Jude Children's Research Hospital, Memphis, TN to DRP were used for all experiments. A seed stock of MDCK cells at passage 23 was subsequently amplified and maintained in Minimal Essential Medium (Gibco) supplemented with 10% fetal bovine serum (FBS; Atlanta Biologicals) and Normocin (Invivogen). 293T cells (ATCC, CRL-3216) were maintained in Dulbecco's Minimal Essential Medium (Gibco) supplemented with 10% FBS and Normocin. All cells were cultured at 37C and 5% CO2 in a humidified incubator.
Authentication	None of the cell lines were authenticated
Mycoplasma contamination	Cell lines were routinely tested for mycoplasma. When tests revealed mycoplasma contamination, the affected cells were replaced by thawing fresh, mycoplasma-free cells.
Commonly misidentified lines<br>(See ICLAC register)	No commonly misidentified cell lines were used.

Animals and other research organisms

Policy information about studies involving animals; ARRIVE guidelines recommended for reporting animal research, and Sex and Gender in Research

Laboratory animals	Guinea pigs (Cavia porcellus, Hartley strain, female at 6 weeks old); Ferrets (Mustela putorius furo, female at 20 weeks old); Swine (Sus scrofa, male & female at 4 weeks old)
Wild animals	Our study did not involve wild animals
Reporting on sex	Female guinea pigs and ferrets were used. A mix of male and female swine were used in the study.
Field-collected samples	Our study did not involve field collected samples
Ethics oversight	Institutional Animal Care and Use Committee (IACUC) of the University of Georgia (ferrets), Emory University (guinea pigs) and Kansas State University (swine) provided oversight for animal experiments.

Note that full information on the approval of the study protocol must also be provided in the manuscript.
